# YC-1 induces lipid droplet formation in RAW 264.7 macrophages

**DOI:** 10.1186/s12929-016-0218-7

**Published:** 2016-01-15

**Authors:** Leo Tsui, Shwu-Fen Chang, Hsiang-Po Huang, Tsorng-Harn Fong, I-Jong Wang

**Affiliations:** Department of Ophthalmology, National Taiwan University Hospital, Taipei, 100 Taiwan; Graduate Institute of Medical Sciences, College of Medicine, Taipei Medical University, Taipei, Taiwan; Graduate Institute of Medical Genomics and Proteomics, College of Medicine, National Taiwan University, Taipei, Taiwan; Department of Anatomy, School of Medicine, College of Medicine, Taipei Medical University, Taipei, 110 Taiwan

**Keywords:** Macrophages, Foam cells, Oxidized low density lipoproteins, YC-1, cGMP-dependent protein kinase

## Abstract

**Background:**

3-(5'-Hydroxymethyl-2'-furyl)-1-benzylindazole (YC-1) is a potential anticancer drug that may activate soluble guanylyl cyclase (sGC) and increase the level of cyclic guanosine monophosphate (cGMP). The aim of this study was to explore the effects of YC-1 on lipid droplet accumulation and foam cell formation in macrophages.

**Results:**

Human-oxidized low density lipoprotein (ox-LDL) was used to induce accumulation of lipid droplets in a murine macrophage cell line, RAW 264.7. Oil red O staining showed that treatment with 20 μM YC-1 for 24 h increased the area of intracellular lipid droplets in macrophages. The results of high content screening (HCS) with the AdipoRed™ assay further revealed that YC-1 enhanced ox-LDL-induced foam cell formation. This was evidenced by an increase in the total area of lipid droplets and the mean fluorescence intensity per cell. Inhibition of cGMP-dependent protein kinase (PKG) using KT5823 significantly reduced YC-1-enhanced lipid droplet formation in ox-LDL-induced macrophage foam cells.

**Conclusion:**

YC-1 induces lipid droplet formation in macrophages, possibly through the sGC/cGMP/PKG signaling pathway. This chemical should be tested with caution in future clinical trials.

## Background

Derived from monocytes, macrophages display tissue-specific phenotypes in different locations of the human body [1]. Macrophages regulate various aspects of normal physiology, including development, tissue homoeostasis, remodeling and repair [2]. Several reports have shown that these cells are also involved in pathological situations, especially in the initiation of the immune response, the engulfing of pathogens and the progression of atherosclerosis [3].

During atherosclerosis development, low density lipoprotein (LDL) in the plasma penetrates the artery wall and becomes oxidized to oxidized-LDL (ox-LDL), which is phagocytosed by macrophages, leading to the formation of foam cells from macrophages [4]. Accumulation of LDL in the intima of blood vessels promotes the migration of circulating monocytes to the endothelial layer and their differentiation into macrophages upon adhesion to the endothelial layer [5]. Subsequently, macrophages engulf excessive intimal lipids via several scavenger receptors [6], becoming foam cells, which have abundant membrane-bound lipid droplets in their cytoplasm [7]. Finally, the cell debris of macrophage foam cells forms the atherosclerotic plaques. Hence, it is reasonable to consider that inhibiting macrophage-derived foam cell formation may be a possible target for therapeutic intervention in atherosclerosis [5, 8].

Recently, we have reported that YC-1 can inhibit oleate-induced lipid accumulation in macrophages [9]. YC-1 is a chemically-synthetic benzylindazole compound, which directly activates soluble guanylyl cyclase (sGC), elevates the level of cyclic guanosine monophosphate (cGMP) in rabbit platelets, and possesses antiplatelet properties [10]. Subsequently, elevated cGMP activates cGMP-dependent protein kinase (PKG) and downstream signal transduction to regulate many cellular responses [11]. Studies have demonstrated several effects of YC-1 on macrophages, including its ability to potentiate the release of tumor necrosis factor-α and nitric oxide production in alveolar macrophages [12, 13]. It has also been found that YC-1 can increase the level of cGMP and inhibit the expression of inducible nitric oxide synthase in RAW 264.7 macrophages [14]. However, the impact of YC-1 on ox-LDL-induced macrophage foam cell formation has not been addressed before. The purpose of this study was to clarify the effect of YC-1 on lipid droplet formation. We hypothesized that YC-1 may regulate lipid droplet and ox-LDL-induced foam cell formation of macrophages through the cGMP signaling pathway. To prove our hypothesis, we used high content screening (HCS), a powerful tool in biological and pharmacological research, to quantitatively detect the influence of YC-1 on the changes in lipid droplets.

## Methods

### Cell culture of RAW 264.7 macrophages

A murine macrophage cell line, RAW 264.7, was purchased from the American Type Culture Collection (Manassas, VA) and cultured, as described in an earlier study [15]. In brief, RAW 264.7 macrophages were maintained in Dulbecco’s Modified Eagle Medium (Invitrogen Life Technologies, Carlsbad, CA) containing 10 % fetal bovine serum (Invitrogen), 100 unit/mL of penicillin (Invitrogen), and 100 μg/mL of streptomycin (Invitrogen) at 37 °C in a humidified incubator with 5 % CO_2_.

### Reagent preparation

YC-1 (Sigma-Aldrich) was dissolved in dimethyl sulfoxide (DMSO; Sigma-Aldrich). KT5823 (Sigma-Aldrich) was dissolved in ethyl acetate (Kanto Chemical Corporation, Tokyo, Japan).

### LDL oxidation

Commercial purified human LDL (Millipore, Temecula, CA) was diluted to 0.5 mg/mL with phosphate-buffered saline (PBS; 137 mM NaCl, 2.7 mM KCl, 8 mM Na_2_HPO_4_, and 1.5 mM KH_2_PO_4_; pH 7.4) and oxidized with 10 μM CuSO_4_ (Sigma-Aldrich) at 37 °C for 24 h [16]. Finally, 1 mM ethylenediaminetetraacetic acid (Sigma-Aldrich) was used to stop the oxidation. After oxidation, the ox-LDL was stored at 4 °C.

### Oil red O staining

Oil red O powder (Sigma-Aldrich) was dissolved in 2-propanol (0.5 %; Kanto Chemical Corporation). The stock was then diluted to 0.3 % oil red O solution with distilled H_2_O and filtered through a 0.22-μm filter. RAW 264.7 macrophages were seeded in a 6-well plate (3 × 10^5^ cells/well; total 2 mL) overnight. After treatment, the treated cells were washed with PBS briefly, and 1 mL of 0.15 % glutaraldehyde (Sigma-Aldrich) was added to each well for 10 min. The fixed cells were washed with PBS three times and stained with 0.5 mL 0.3 % oil red O solution for 5 min. Finally, the stained cells were washed with PBS three times and observed with a Nikon Eclipse E600 microscope (Nikon Instruments Inc., Tokyo, Japan) and an Evolution™ MP digital camera (Media Cybernetics, Bethesda, MD). All experiments were repeated three times, and the representative data are shown.

### Cell viability assay

3-(4,5-Dimethylthiazol-2-yl)-2,5-diphenyltetrazolium bromide (MTT) powder (Sigma-Aldrich) was dissolved to 5 mg/mL with distilled H_2_O. The MTT stock solution was sterilized through a 0.22-μm filter and stored at 4 °C. RAW 264.7 macrophages were seeded in a 24-well plate (10^4^ cells/well; total 0.5 mL) overnight and treated until 80 % confluency. After treatment, 50 μL of MTT stock solution was added to each well and incubated with 5 % CO_2_ at 37 °C for 2 h. Finally, the medium was removed and 0.5 mL of 2-propanol was added to each well. After 5 min at room temperature, 200 μL of solution from each well was transferred to a 96-well plate and measured at 590 nm by a Multiskan RC microplate reader (Thermo LabSystems, Helsinki, Finland). All experiments were repeated three times.

### Quantification of total lipid content

Quantification of total lipid content was measured based on a previously-published protocol [17]. In brief, RAW 264.7 macrophages were seeded in a 24-well plate (10^4^ cells/well; total 0.5 mL) overnight and treated until 80 % confluency. After treatment, the cells were stained with oil red O, and the intracellular lipid was extracted by adding 200 μl DMAO to each well. After 5 min at room temperature, 200 μL of solution from each well was transferred to a 96-well plate. Absorbance was measured at 510 nm with a μQuant microplate reader (BioTek Instruments, Winooski, VT). The quantified results were corrected after parallel experiments of cell viability assay. All experiments were repeated three times.

### Measurement of intracellular cGMP

RAW 264.7 macrophages were seeded in a 24-well plate (10^4^ cells/well; total 0.5 mL) overnight and treated until 80 % confluency. After treatment, intracellular cGMP of macrophages was measured using cGMP Enzymeimmunoassay Biotrak System (Amersham Biosciences, Little Chalfont Buckinghamshire, UK). Absorbance was measured at 450 nm with a μQuant microplate reader (BioTek Instruments, Winooski, VT). All experiments were repeated three times.

### HCS with AdipoRed™ assay

Quantification of lipid storage compartments in macrophages and ox-LDL-induced foam cells was performed, based on a previously-published protocol [18]. RAW 264.7 macrophages were seeded in a Costar® 96-well black solid plate (3000 cells/well; total 100 μL; Bio-Rad Laboratories, Hercules, CA). After treatment, the cells were stained with AdipoRed™ (1:40; Lonza Walkersville, Inc., Walkersville, MD) and Hoechst 33342 (1:1000; Invitrogen) for 0.5 h. The sample was analyzed by a Thermo Scientific Cellomics® ArrayScan® VTI HCS Reader (Thermo Fisher Scientific, Pittsburgh, PA), and the Columbus™ Image Data Storage. Analysis System software (PerkinElmer, Columbus, OH) was used to recolor the images and perform the HCS image analysis. The intracellular lipid droplets were analyzed using the Spot Detector protocol of the Cellomics® HCS Reader. All HCS experiments were repeated at least three times.

### Statistical analysis

All data are presented as the mean ± standard deviation. The statistical significance of differences between groups was analyzed using one-way analysis of variance with Tukey’s post hoc test by SAS version 9.4 (SAS Institute, Cary, NC). Differences with a *P* value less than 0.05 were considered significant.

## Results

### YC-1 induces lipid droplet accumulation in RAW 264.7 macrophages

**Fig. 1:**
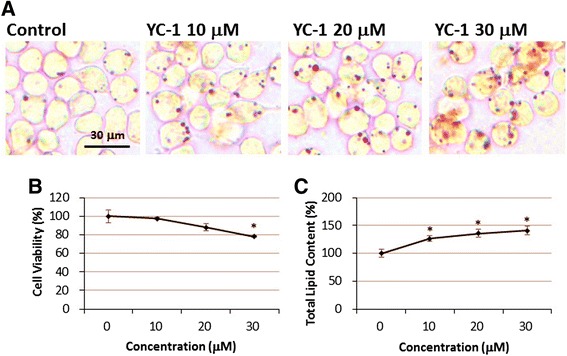
The effect of different doses of YC-1 on lipid droplet formation and cell viability in RAW 264.7 macrophages. **a** Dosage-dependent YC-1 enhanced lipid droplet formation. RAW 264.7 macrophages were treated with different concentrations of YC-1 for 24 h. After fixation and staining with oil red O, the cells were observed by light microscopy. Scale bar = 30 μm. **b** 30 μM YC-1 reduced the cell viability of macrophages. RAW 264.7 macrophages were treated with different concentrations of YC-1 for 24 h and the cell viability was measured by the MTT assay (*n* = 6). **c** Quantification of total lipid content of macrophages (*n* = 6). * indicates *P* < 0.05 compared to the control group

**Fig. 2:**
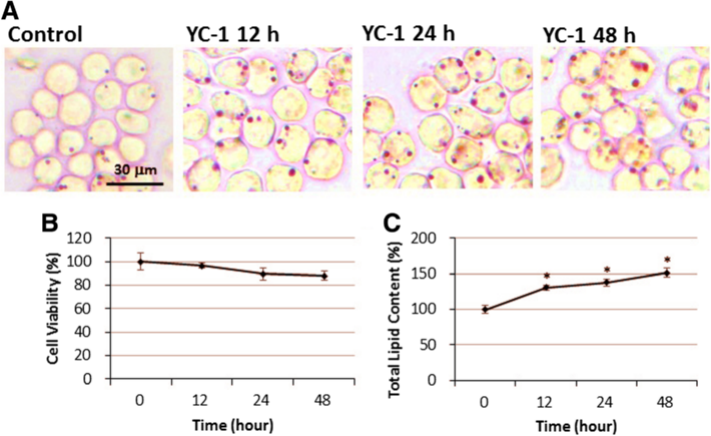
Time course effect of YC-1 on lipid droplet formation and cell viability in RAW 264.7 macrophages. **a** Time course analysis of YC-1-induced lipid droplet formation in macrophages. RAW 264.7 macrophages were treated with 20 μM YC-1 for the indicated lengths of time. After fixation and staining with oil red O, the cells were observed by light microscopy. Scale bar = 30 μm. **b** Time course analysis of the cell viability of YC-1-treated macrophages. RAW 264.7 macrophages were treated with 20 μM YC-1 for different lengths of time and the cell viability was measured by MTT assay (*n* = 6). **c** Quantification of total lipid content of macrophages (*n* = 6). * indicates *P* < 0.05 compared to the control group

To analyze the effect of YC-1 on lipid droplet formation, we treated RAW 264.7 macrophages with YC-1 for different lengths of time (12, 24 and 48 h) and with different doses (10, 20 and 30 μM). We found that macrophages treated with 10, 20, and 30 μM YC-1 for 24 h displayed a significant increase in the area of intracellular lipid droplets and total lipid content compared to the control group, as detected and quantified by oil red O staining (Fig. 1a and c). We also found that macrophages treated with 20 μM YC-1 for 12, 24 or 48 h displayed a significant increase in lipid droplet accumulation (Fig. 2a and c). In addition, the result of the MTT cell viability assay revealed that YC-1 did not significantly affect cell survival except with doses of 30 μM (or higher) used for 24 h (Figs. 1b and 2b). These results demonstrate that YC-1 stimulates lipid droplet formation in macrophages and suggests that YC-1 may affect foam cell formation in macrophages.

### YC-1 induces foam cell formation in RAW 264.7 macrophages

**Fig. 3:**
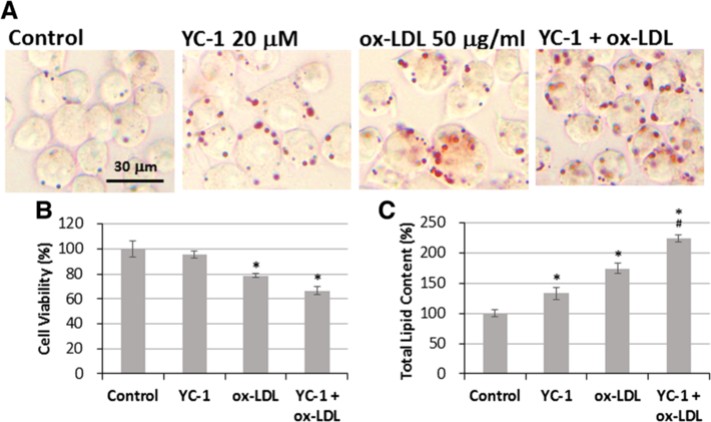
Treatment with YC-1 enhances ox-LDL-mediated foam cell formation. **a** YC-1 increased intracellular lipid droplets in ox-LDL-treated macrophages. RAW 264.7 macrophages were treated with 20 μM YC-1 with/without 50 μg/mL ox-LDL for 24 h. After fixation and staining with oil red O, the cells were observed by light microscopy. Scale bar = 30 μm. **b** Treatment with ox-LDL decreased the cell viability of macrophages. RAW 264.7 macrophages were treated with 20 μM YC-1 with/without 50 μg/mL ox-LDL for 24 h and the cell viability was measured by MTT assay (*n* = 6). **c** Quantification of total lipid content of macrophages (*n* = 6). * indicates *P* < 0.05 compared to the control group; # indicates *P* < 0.05 compared to the ox-LDL-treated group

To further evaluate the effect of YC-1 on foam cell formation, commercial LDL was oxidized with copper ions and was then used to induce the formation of lipid-laden macrophages. The results of oil red O staining show that treatment with 50 μg/mL ox-LDL for 24 h induced lipid droplet formation (Fig. 3a) but significantly decreased cell viability in macrophages (Fig. 3b). We also observed that YC-1 enhanced ox-LDL-induced lipid droplet formation. The quantification result revealed that co-treatment with ox-LDL and 20 μM YC-1 for 24 h significantly increased total lipid content compared to treatment with ox-LDL alone (Fig. 3c). This result indicates that YC-1 may enhance ox-LDL-induced foam cell formation in macrophages.

**Fig. 4:**
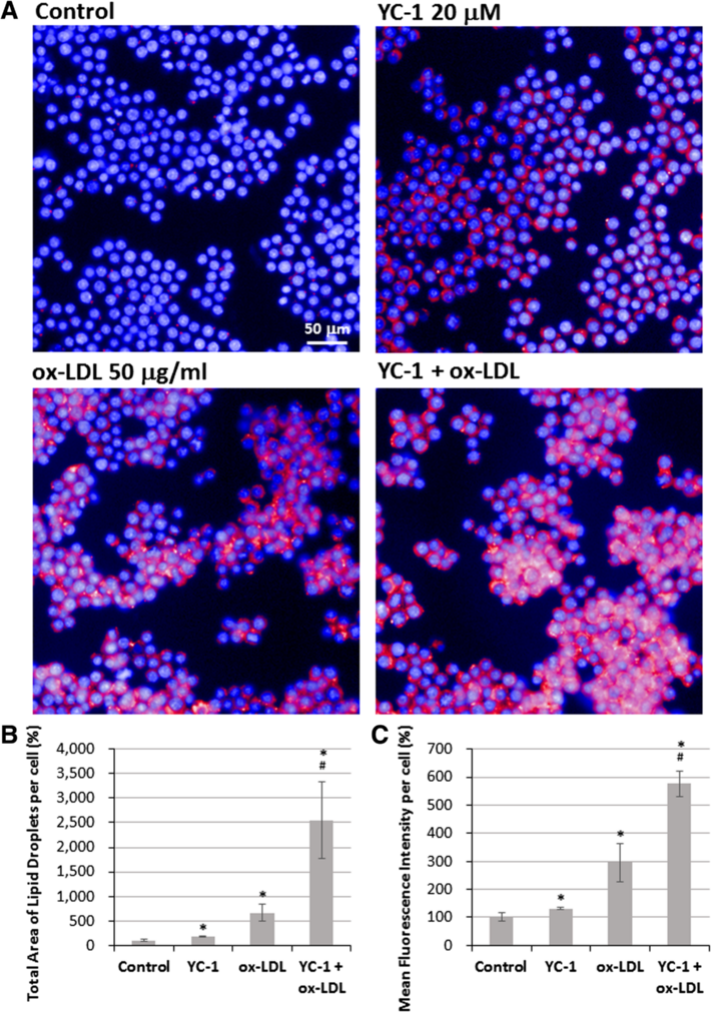
YC-1 induces lipid droplet formation in ox-LDL-mediated foam cells. RAW 264.7 macrophages were treated with 20 μM YC-1, 50 μg/mL ox-LDL, or both YC-1 and ox-LDL for 24 h. After fluorescence staining with AdipoRed™ (red for intracellular lipid) and Hoechst 33342 (blue for nucleus), the total area of lipid droplets and mean fluorescence intensity labelled by AdipoRed were detected and analyzed in each cell using an HCS reader. Scale bar = 50 μm. **a** The imaging of HCS. **b** Quantification of total area of lipid droplet per cell (*n* = 5). **c** Quantification of mean fluorescence intensity per cell (*n* = 5). * indicates *P* < 0.05 compared to the control group; # indicates *P* < 0.05 compared to the ox-LDL-treated group

To further confirm the effect of YC-1 on lipid droplet and foam cell formation, we used the AdipoRed™ assay to label lipid accumulation in macrophages. HCS was used to quantify the total area of lipid droplets and mean fluorescence intensity in individual cells to estimate the level of intracellular lipid accumulation. The results of the HCS image analysis show that YC-1 increased the amount of intracellular lipid compared to the control group, and enhanced ox-LDL-induced lipid accumulation in macrophages compared to the ox-LDL-treated group (Fig. 4a). Compared with the control cells, YC-1 increased the total area of lipid droplets by 75 % and the mean fluorescence intensity per cell by 30 % (Fig. 4b and c). Moreover, YC-1 significantly increased the total area of lipid droplets (by 1900 %) and the mean fluorescence intensity (by 275 %) in ox-LDL-induced lipid droplets compared to the ox-LDL-treated group (Fig. 4b and c). These results further confirm that YC-1 promotes ox-LDL-induced foam cell formation in macrophages.

### PKG inhibitor decreases YC-1-induced lipid droplet accumulation and foam cell formation in macrophages

**Fig. 5:**
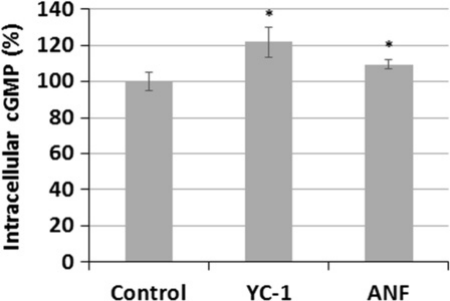
YC-1 and ANF increases the level of intracellular cGMP in macrophages. RAW 264.7 macrophages were treated with 20 μM YC-1 or 10 μg/mL ANF for 16 h. The intracellular cGMP was measured using a cGMP Enzymeimmunoassay kit (*n* = 5). * indicates *P* < 0.05 compared to the control group

**Fig. 6:**
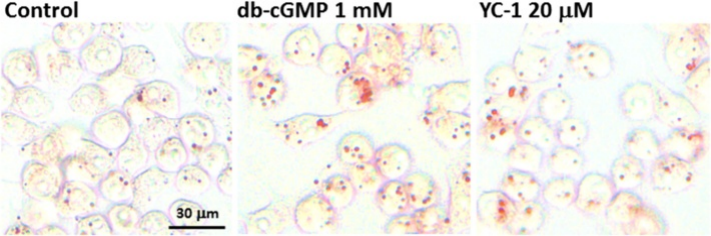
YC-1 and db-cGMP induce lipid droplet formation in RAW 264.7 macrophages. RAW 264.7 macrophages were treated with 20 μM YC-1 or 1 mM db-cGMP for 24 h. After fixation and staining with oil red O, the cells were observed by light microscopy. Scale bar = 30 μm

To clarify the involvement of the sGC/cGMP/PKG signaling pathway in YC-1-induced lipid accumulation, intracellular cGMP was measured in macrophages. The sGC activator, atrial natriuretic factor (ANF), was used as a positive control [19]. We found that treatment with 20 μM YC-1 for 16 h, like the treatment of the positive control ANF, significantly increased the level of cGMP (Fig. 5). The result of oil red O staining also revealed that the cGMP analogue dibutyryl cyclic guanosine monophosphate (db-cGMP) induced lipid droplet formation in macrophages in a manner similar to that obtained with YC-1 (Fig. 6). These findings support the idea that YC-1 may regulate lipid accumulation in macrophages through the cGMP-related pathway.

**Fig. 7:**
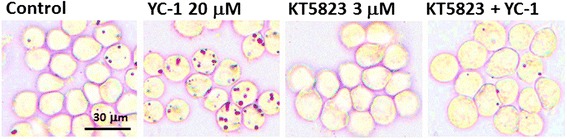
The PKG inhibitor KT5823 inhibits YC-1-induced lipid droplet formation in RAW 264.7 macrophages. RAW 264.7 macrophages were treated with 20 μM YC-1, 3 μM KT5823 or both of YC-1 and KT5823 for 24 h. After fixation and staining with oil red O, the cells were observed by light microscopy. Scale bar = 30 μm

**Fig. 8:**
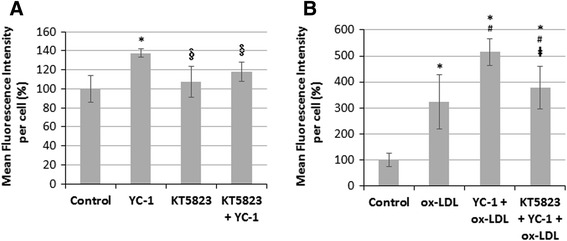
PKG inhibitor reduces YC-1-stimulated lipid accumulation in macrophages and ox-LDL-mediated foam cells. **a** PKG inhibitor reduced YC-1-induced lipid accumulation in macrophages. RAW 264.7 macrophages were treated with or without 3 μM KT5823, and then exposed to 20 μM YC-1 for 24 h. After fluorescence staining with AdipoRed™ and Hoechst, the mean fluorescence intensity (AdipoRed) per cell was detected and analyzed by an HCS reader (*n* = 5). **b** PKG inhibitor reduced YC-1-induced lipid accumulation in ox-LDL-mediated foam cells. RAW 264.7 macrophages were treated with or without 3 μM KT5823, and then exposed to 20 μM YC-1 and 50 μg/mL ox-LDL for 24 h. After fluorescence staining with AdipoRedTM and Hoechst, the mean fluorescence intensity (AdipoRed) per cell was detected and analyzed by an HCS reader (*n* = 5). * indicates *P* < 0.05 compared to the control group; § indicates *P* < 0.05 compared to the YC-1-treated group; # indicates *P* < 0.05 compared to the ox-LDL-treated group; indicates *P* < 0.05 compared to the YC-1 and ox-LDL-treated group

To further clarify the effect of YC-1 on lipid droplet formation in macrophages and foam cells, we used KT5823 to attenuate the activation of PKG [20]. The results of oil red O staining show that KT5823 inhibited YC-1-induced lipid droplet accumulation (Fig. 7), suggesting that YC-1 may induce lipid accumulation in macrophages via the PKG-related signaling pathway. The HCS results revealed that KT5823 significantly inhibited YC-1-induced lipid droplet formation, as the mean fluorescence intensity per cell of KT5823-treated groups was reduced by 20 % (Fig. 8a) and 140 % (Fig. 8b) in macrophages and in ox-LDL-mediated macrophage foam cells, respectively. Our findings suggest that the sGC/cGMP/PKG signaling pathway may be involved in YC-1-induced lipid droplet accumulation and foam cell formation in macrophages.

## Discussion

We have demonstrated that YC-1 increases the number of intracellular lipid droplets and total lipid content in RAW 264.7 macrophages, and enhances ox-LDL-induced foam cell formation. In addition, the results show that the PKG-related signaling pathway is involved in YC-1-regulated lipid droplet formation in macrophages and foam cells.

We have previously reported that YC-1 inhibits oleate-induced lipid droplet formation, leading to lipolysis in lipid-laden macrophages [9]. Oleate, a type of unsaturated free fatty acid, is reported to inhibit cholesterol efflux in modified LDL-treated macrophages [21] and to induce triglyceride accumulation in macrophages [22]. However, ox-LDL is composed not only of triglycerides, but also of cholesterol and cholesterol esters [23–25]. The uptake of ox-LDL is through scavenger receptors and endocytosis in macrophages [26], whereas oleate is probably transported via diffusion [27]. These differences may explain the different effects of YC-1 on the ox-LDL-induced macrophage foam cells used in this study and oleate-induced lipid-loaded macrophages. Therefore, we speculate that YC-1 may regulate scavenger receptor–mediated endocytosis, and subsequently enhance lipid droplet formation in macrophage foam cells. In addition, we have demonstrated here that YC-1 increases the number of intracellular lipid droplets in macrophages as well as in ox-LDL-induced macrophage foam cells. This could be explained by the possibility of YC-1 increasing the clearance of fatty acids in oleate-treated macrophages, while enhancing the uptake of cholesterol or triglycerides supplied by serum or ox-LDL.

We have previously shown that treating cells with YC-1 for 10 min does not affect sGC activity and cGMP levels in RAW 264.7 macrophages [9]. In contrast to this, it has also been reported that treatment of J774A.1 macrophages with YC-1 (20 and 40 μM) for 24 h significantly increases intracellular cGMP [28]. In this study, we showed that YC-1, as well as the sGC activator ANF, increased the level of intracellular cGMP at 16 h. The result of oil red O staining revealed that YC-1 and the cGMP analogue db-cGMP induced lipid droplet formation in macrophages. We also demonstrated that the PKG inhibitor KT5823 inhibits YC-1-induced lipid droplet formation in RAW 264.7 macrophages and foam cells, as evidenced by the reduction of the mean fluorescence intensity (per cell) in KT5823-treated cells. In addition, it seems that a longer treatment with YC-1 (e.g., 24 h) may be required to continually activate the sGC/cGMP/PKG pathway in macrophages and induce lipid droplet and foam cell formation. In addition, previous studies have demonstrated that treatment with 100 μg/ml ox-LDL for 4 h had no impact on the level of cGMP in human monocyte-derived macrophages [29]. However, nitric oxide could regulate apoptosis of macrophages through guanylate cyclase stimulation [29]. According to the study of Chen et al., ox-LDL can decrease intracellular cGMP in human platelets [30]. Similarly, ox-LDL has been reported to activate human platelets through inhibition of the cGMP signaling cascade in the study of Magwenzi et al. [31]. These studies have clearly revealed the possible relationship among ox-LDL, cGMP, and sGC/cGMP/PKG cascades.

HCS, consisting of automated microscopy and image analysis, is a new technology that has been applied to drug discovery and cell biology [32]. It has also been used to identify specific proteins or cellular structures with immunoreagents, organic dyes, genetically-encoded fluorescent proteins or quantum dots [33]. Using the technique of HCS with hydrophobic fluorescence dye to detect intracellular lipid droplets and ox-LDL-induced lipid accumulation in macrophages has been reported in the research literature [18]. To estimate the level of intracellular lipid accumulation, we used HCS and AdipoRed™ assay to label and quantify the total area of lipid droplets and the mean fluorescence intensity per cell. Tsou et al. have shown that YC-1 can decrease cholesterol content in ox-LDL-treated macrophages through activation of sGC, but that the protein level of scavenger receptor class A did not change [28]. In contrast, we used HCS and the AdipoRed™ assay to quantify lipid droplet and foam cell formation in macrophages instead of the cholesterol content assay, as foam cells are defined as being “full of intracellular lipid droplets” in macrophages and smooth muscle cells.

YC-1 is a promising antiangiogenic anticancer agent that functions by targeting HIF-1α and has effectively prevented tumor growth in immunodeficient mice grafted with five types of human tumor cells and in Hep3B cells [34, 35]. Yeo et al. have proposed that YC-1 should be regarded as a good lead compound for the development of novel antiangiogenic and anticancer agents [36]. Our current data suggest that YC-1 enhances ox-LDL-mediated lipid accumulation and foam cell formation, which may in turn contribute to atherosclerosis. Hence, this chemical should be used cautiously in future clinical trials.

## Conclusion

In summary, YC-1 induces lipid droplet formation in macrophages, possibly via an sGC/cGMP/PKG-related pathway.

## Abbreviations

ANF: Atrial natriuretic factor
cGMP: cyclic guanosine monophosphate
db-cGMP: dibutyryl cyclic guanosine monophosphate
HCS: high content screening
MTT: 3-(4,5-cimethylthiazol-2-yl)-2,5-diphenyl tetrazolium bromide
ox-LDL: oxidized low density lipoprotein
PKG: cGMP-dependent protein kinase
sGC: soluble guanylate cyclase
YC-1: 3-(5'-Hydroxymethyl-2'-furyl)-1-benzyl indazole
